# Molecular subtyping of *Blastocystis* sp. detected in patients at a large tertiary referral hospital in Lusaka, Zambia

**DOI:** 10.3389/fpara.2022.1033485

**Published:** 2022-10-27

**Authors:** Gilbert Munsaka, Kyoko Hayashida, Benjamin Mubemba, Edgar Simulundu, Namwiinga Mulunda, Ruth Pule, Sandie Sianongo, Marina Makuluni, Walter Muleya, Katendi Changula, Simbarashe Chitanga, Mable Mutengo

**Affiliations:** ^1^ Department of Pathology and Microbiology, University Teaching Hospitals, Lusaka, Zambia; ^2^ Division of Collaboration and Education, International Institute for Zoonosis Control, Hokkaido University, Sapporo, Japan; ^3^ International Collaboration Unit, International Institute for Zoonosis Control, Hokkaido University, Sapporo, Japan; ^4^ Department of Wildlife Sciences, School of Natural Resources, Copperbelt University, Kitwe, Zambia; ^5^ Department of Biomedical Sciences, School of Medicine, Copperbelt University, Ndola, Zambia; ^6^ Macha Research Trust, Choma, Zambia; ^7^ Department of Disease Control, School of Veterinary Medicine, University of Zambia, Lusaka, Zambia; ^8^ Department of Biomedical Sciences, Kamuzu University of Health Sciences, Blantyre, Malawi; ^9^ Department of Biomedical Studies, School of Veterinary Medicine, University of Zambia, Lusaka, Zambia; ^10^ Department of Paraclinical Studies, School of Veterinary Medicine, University of Zambia, Lusaka, Zambia; ^11^ Department of Pathobiology, School of Veterinary Medicine, University of Namibia, Windhoek, Namibia; ^12^ Department of Biomedical Sciences, School of Health Sciences, University of Zambia, Lusaka, Zambia; ^13^ School of Life Sciences, University of KwaZulu-Natal, Durban, South Africa; ^14^ Institute of Basic and Biomedical Sciences, Levy Mwanawasa Medical University, Lusaka, Zambia

**Keywords:** *Blastocystis*, subtyping, 18S rRNA, phylogenetics, Zambia

## Abstract

**Background:**

*Blastocystis* sp. is a common enteric eukaryote of humans whose pathogenicity is still debatable. However, a number of reported *Blastocystis* colonization associated with enteric disease exist. In Zambia, presence of the pathogen has previously been reported in children. However, the molecular epidemiology of *Blastocystis* colonization remains unclarified in Zambia.

**Methods and results:**

Archived stool samples submitted for routine parasitological diagnosis at Zambia’s largest tertiary referral hospital positive for *Blastocystis* sp. by microscopic examination were selected for the study. Subtyping of the *Blastocystis* was done based on polymerase chain reactions (PCR) amplification, sequencing and subsequent phylogenetic analysis of the 18S small subunit (*SSU*) rDNA gene. Four subtypes, ST1 (allele 4), ST2 (allele 12), ST3 (allele 34, 36, 37, 38, 39) and ST6 (allele 122), were identified by molecular procedures in the study, with some Zambian sequences showing close relationships with those detected in non-human primates and common rats.

**Conclusions:**

The study revealed the circulation of multiple *Blastocystis* subtypes ST1, 20% (9/45), ST2, 15% (7/45), ST3 24.4% (11/45), and ST6, 2.2% (1/45) in the study population. The close clustering of some Zambian sequences with those detected from animals suggests the possibility of the presence of both anthroponotic and zoonotic transmission cycles in the country. Further studies in animal populations are recommended for a better understanding of the epidemiology of *Blastocystis* and for implementation of effective evidence-based control strategies.

## Introduction

The Blastocystis sp. is a single-celled anaerobic eukaryote of the Stramenopile group, commonly found in the enteric system of most mammals (Clark et al., 2013). Colonization occurs through the faecal–oral route. The parasite has a worldwide distribution and is estimated to be colonizing about one billion people globally (Scanlan and Stensvold, 2013). Whilst the exact pathogenicity of the parasite to humans is still undetermined, there have been reports associating parasite presence with irritable bowel syndrome (Tan et al., 2010; Jimenez-Gonzalez et al., 2012

Additionally, extra-intestinal manifestations, including urticaria (Bahrami et al., 2019). with potential of severe disease in immunocompromised individuals (Lepczyńska et al., 2017).

In the past, the designation of species for Blastocystis was based on host range, and this caused confusion. However, it was resolved with the agreed nomenclature that used the Blastocystis sp. subtype (ST) designation (Stensvold et al., 2007; Maloney and Santin, 2021), based on the sequencing of the small subunit ribosomal DNA (SSU-rDNA). It has been established that it is not necessary to amplify the whole SSU-rDNA gene in order to allocate a sample to a known subtype (Clark et al., 2013), with whole gene sequencing only indicated if a sample doesn’t fit into any of the previously established subtypes. Currently, the standard sets of primers are available for the SSU rRNA locus of Blastocystis isolates, including those from Scicluna et al. (2006) and Santín et al., 2011. As such, a number of diagnostic regions of the SSU rDNA have been used by different authors (Stensvold et al., 2006; Parkar et al., 2010), and based on this, a total of 32 subtypes are currently recognized in mammalian and avian hosts (Santín et al., 2011; Maloney et al., 2018; Maloney et al., 2019; Maloney et al., 2021; Higuera et al., 2021; Hublin et al., 2021); Baek et al., 2022).

Of the 32 Blastocystis subtypes reported, ST1 – 9 and ST12 have been reported in humans (Alfellani et al., 2013; Clark et al., 2013; Ramírez et al., 2016). However, it has been shown that these subtypes are not specific for humans as they have also been reported in other mammals and birds, a finding which supports the idea of the existence of zoonotic or reverse zoonotic transmission of the parasite (Cian et al., 2017; Hublin et al., 2021). This assertion is further supported by the sporadic detection in humans of subtypes (ST10, ST14, and ST16) that are typically isolated from animals (Khaled et al., 2020; Osorio-Pulgarin et al., 2021). The possibility of zoonotic transmission playing a significant role in the epidemiology of the parasite is further supported by the observed relatively high prevalence amongst populations that closely associate with animals (Rajah et al., 1999; Rivera, 2008; Stensvold et al., 2009; Yoshikawa et al., 2009; Parkar et al., 2010; Wang et al., 2014; Yoshikawa et al., 2016; Greige et al., 2018). As such, subtype analysis amongst different hosts within the same geographical area can shed light on transmission of the commensal between the different host species (Andersen et al., 2016).

With the use of molecular tools, Blastocystis colonization is frequently reported, including new subtypes. Across the globe, reported prevalence varies widely, with high prevalence rates being reported in areas of low hygiene (Kevin, 2008). Globally, the reported prevalence of Blastocystis colonization in human populations ranges from as low as 5% to as high as 100% (Safadi et al., 2014; El et al., 2016; Raul et al., 2019). In Africa, a number of countries have reported varying colonization rates of Blastocystis in humans, with subtypes 1,2, 3, 4, 5, 6 and 7 being frequently reported, along with mixed infections involving different subtypes (Alfellani et al., 2013; Safadi et al., 2014; Elena et al., 2018). In Zambia, Blastocystis colonization was previously reported in children from one rural community, with a prevalence of 53.8% (Graczyk et al., 2005) as well as in both hospitalised and nonhospitalised HIV patients with prevalence rate of 21% (Hunter et al., 1992). The results from these studies were based on microscopy and there has never been a report on the subtypes circulating in the country. Here, we aimed to subtype the Blastocystis parasite using archived stool samples that were submitted for routine parasitological examination at the University Teaching Hospitals, Zambia’s largest tertiary referral hospital.

## Materials and methods

### Study area and sample collection

Samples used in this study were archival samples that were collected from patients who presented at the University Teaching Hospitals, in Lusaka and were positive for Blastocystis sp. on microscopic examination on initial screening. This is a national referral hospital with patients coming from all the regions of the country.

### DNA extraction

The DNA was extracted from Blastocystis microscopic positive samples (N=85) using the fecal/soil Microbe

Miniprep Kit Quick-DNA™ (Zymo Research, Orange, CA, USA) in accordance with the manufacturer’s recommendations. The extracted DNA was then used in subsequent PCR.

### PCR amplification and sequencing

Genomic DNA was subjected to PCR analysis using primer pair BhRDr and RD5, targeting a 600bp fragment of the 18S SSU-rDNA gene of Blastocystis (Scicluna et al., 2006). Briefly, the amplification was done in a 10 µL volume reaction mixture containing; 2.55 µL of nuclease-free water, 5 µL of 2x Ampidirect (Shimadzu, Kyoto, Japan), 0.2 µL of 10 µM each primer (final 0.2 µM), 0.05 µL of BIO-TAQ HS (5U/µl; Bioline, London, UK), and 2 µL of DNA. Amplification conditions were with an initial denaturation step at 94°C for 10 min, followed by 40 cycles at 94°C for 30 s, 55°C for 60 s, and 72°C for 1 min. Known positive Blastocystis DNA was used as positive control, whilst nuclease-free water was used as a no template control. PCR products were electrophoresed on 1.2% agarose gel stained with GelRed (Biotium, Hayward, CA, USA) and visualized under ultraviolet (UV) light.

For sequencing, PCR products were purified using Wizard® SV Gel and Clean-Up System (Promega, Madison, WI, USA). Bidirectional Sanger sequencing was conducted with the purified amplicons as a template using a Big Dye™ Terminator Cycle Sequencing Kit v3.1 (Applied Biosystems, Carlsbad, CA, USA) according to the manufacturer’s protocol. Nucleotide sequences were assembled and edited using GENETYX ATGC software version 7.5.1 (GENETYX Corporation, Tokyo, Japan). The obtained nucleotide sequences were then deposited in GenBank under the following accession numbers: LC639128 – LC639172. Furthermore, we used the online PubMLST tool (https://pubmlst.org/bigsdb?db=pubmlst_blastocystis_seqdef&page=sequenceQuery) to assign sequences generated in the present study the subtypes and explore their genomic diversity alleles (Jolley et al., 2018).

The partial sequences of the 18S SSU-rDNA gene of Blastocystis sp. generated in this study, together with selected reference sequences in GenBank were used for phylogenetic analysis to produce maximum likelihood (ML) trees. Firstly, Zambian sequences were analysed alone and based on the resulting topology of this initial phylogenetic tree, a representative number of isolates (N=28) were selected so that each clade was represented when included in the analysis with reference sequences obtained from GenBank. The BLAST (https://blast.ncbi.nlm.nih.gov/) searching tool was also used to identify the closest subtypes to the sequences generated in this study to those that have been characterised and are available in GenBank. For phylogenetic analysis, sequences were aligned using the multiple sequence alignment program MAFFT (online version 7) with the alignment strategy set to FFT-NS2 (Katoh et al., 2019). The sequence of Proteromonas laccertae (GenBank accession: U37108.1) was included as outgroup in the alignment. Resulting Fasta alignments were then reformatted to a Phylip alignment using SeaView v4 (Gouy et al., 2010). Phylogenetic inference was then performed on the resulting final alignment using the online ATGC PhyML-SMS tool (http://www.atgcmontpellier.fr/phyml-sms/). The phylogenetic tree was constructed by the ML method using smart model selection (Lefort et al., 2017) employing a Bayesian Information Criterion (BIC) for model selection. For tree improvement, subtree pruning and regrafting (SPR) was selected, otherwise using default settings. Branch support was evaluated using Shimodaira-Hasegawa approximate likelihood ratio test (SH-like aLRT). The resultant maximum likelihood tree files were then edited using iTOL (https://itol.embl.de/) (Letunic and Peer, 2019).

## Results

From 85 microscopically positive samples that were subjected to subtyping, 71 were Blastocystis positive by PCR from which we managed to obtain 45 good consensus sequences that allowed us to detect different subtypes and alleles (**Supplementary Table 1**). When compared to sequences in GenBank using the basic alignment search tool (BLAST) (https://blast.ncbi.nlm.nih.gov/Blast.cgi), Blastocystis 18S SSU-rDNA nucleotide sequences obtained from sequenced samples showed sequence identity ranging from 98.5 – 100% (**Supplementary Table S2**). In addition, Zambian isolates shared sequence identity ranging from 37.1%-100% (**Supplementary Table S3**).

**Figure 1:**
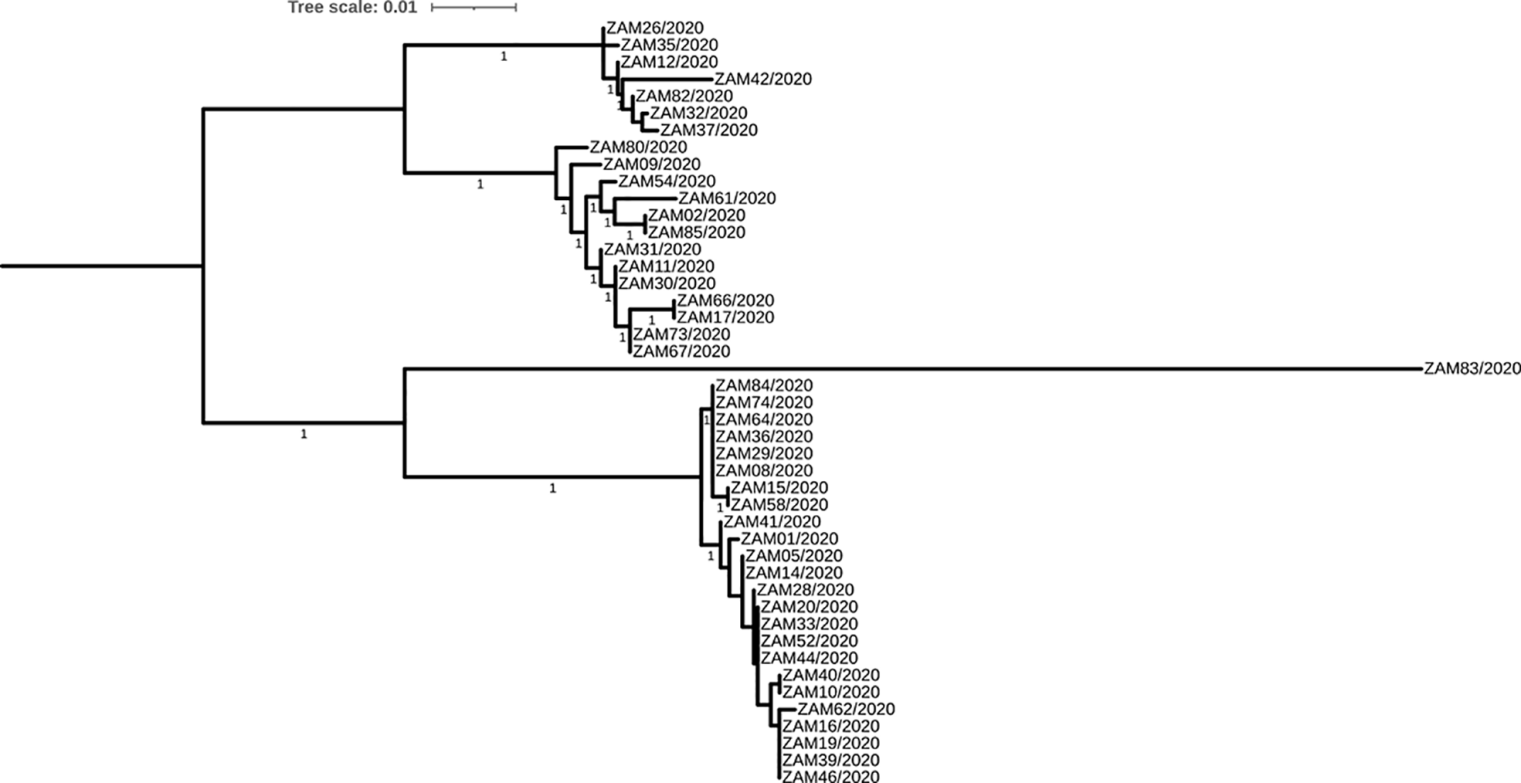
Maximum likelihood (ML) tree showing phylogenetic relationships of 45 sequences of Blastocystis sp. detected in human-derived samples in Zambia on partial sequences of the small subunit rRNA gene. Branches supported by SH-like aLRT values above 0.95 are indicated by the values next to the branches they represent. Bar, number of nucleotide substitutions per variable site.

**Figure 2:**
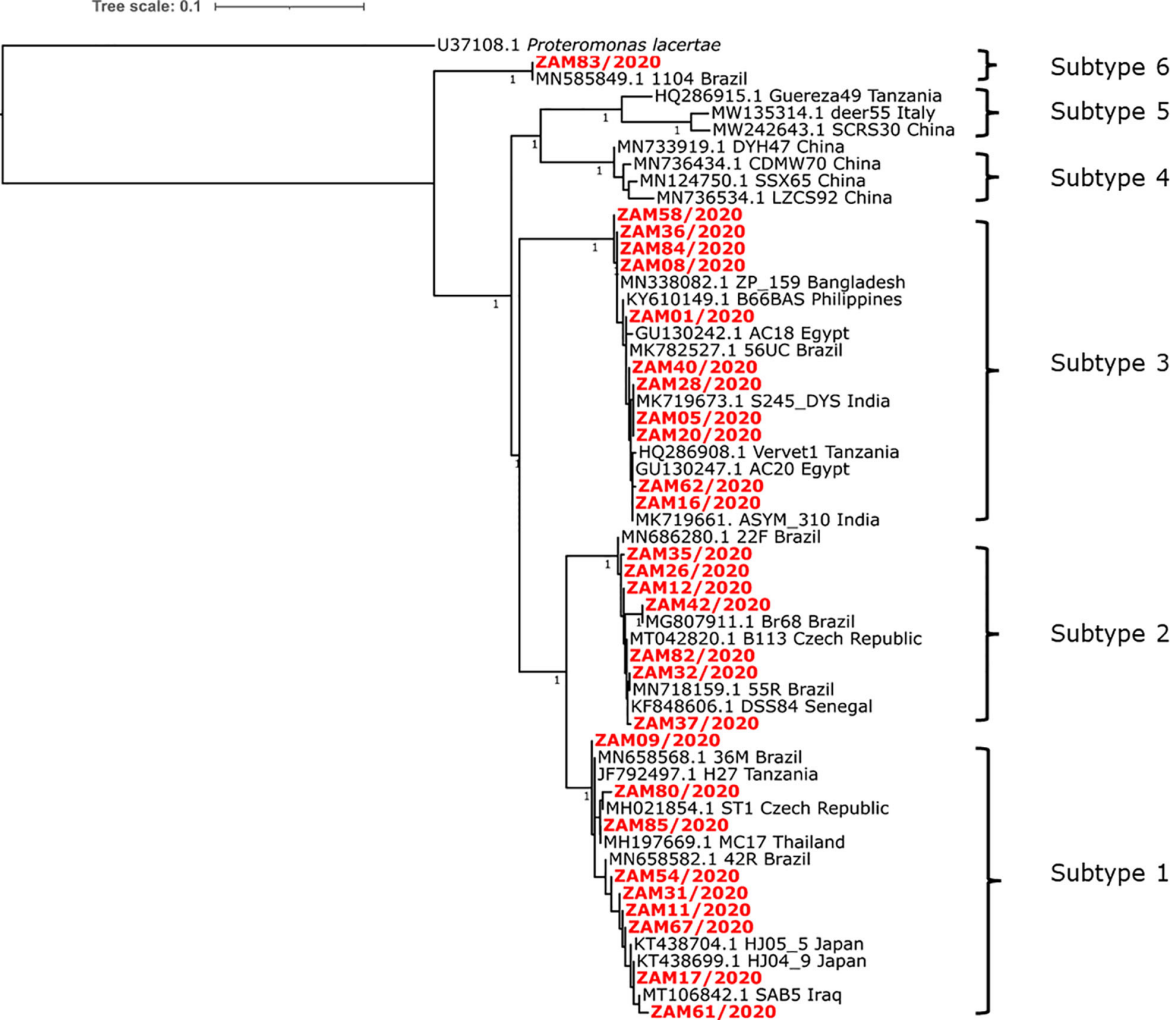
Maximum likelihood (ML) tree showing phylogenetic relationships of selected Blastocystis sp .(n=28) detected in Zambia as well as reference sequences obtained from GenBank (n=29). All Zambian derived isolates sequenced in this study are indicated in bold and are in red text at the tip labels while for reference sequences, the tip labels show their GenBank accession number, isolate ID and the country of origin. Branches supported by SH like aLRT values above 0.95 are indicated by the values next to the branches. Bar, number of substitutions per variable site.

When analysed alone, phylogenetic analysis of sequences generated in this study yielded an ML tree with four distinct monophyletic groups that were statistically supported, suggesting that there were at least four subtypes of Blastocystis sp. detected in the human-derived samples tested (**Figure 1**). Topologically, the phylogenetic ML tree of sequences generated from this study and those from GenBank showed the separation of the sequences into six subtypes (1-6) (**Figure 2**). The Zambian sequences segregated into four subtypes, namely ST1 (allele 4), ST2 (allele 12), ST3 (allele 34, 36, 37, 38, 39) and ST6 (allele 122) (**Supplementary Table S1**), and were closely related to sequences from various continents including Africa, South America, Europe and Asia (**Figure 2**). In addition, with regards to genomic diversity of the subtypes based on allele assignment, the detected subtypes exhibited no intrasubtype genetic heterogeneity except for subtype ST3 where several alleles were detected. While there were several sequences from Zambia in subtypes ST1-3, only one sequence belonged to subtype ST6 and was identical to sequence (GenBank accession: MN585849.1) obtained from a human in Brazil. Interestingly, Zambian sequences that segregated into subtype ST1 and ST3 clusters, showed close relationships with sequences obtained from a common brown rat (experimental colonization using human isolate) and a nonhuman primate, respectively (**Figure 2**).

## Discussion

To the best of our knowledge, this is the first study to characterize subtypes circulating in Zambia. From the samples which were subtyped, there was a predominance of ST3, which agrees with existing knowledge that this is the most commonly isolated ST in humans and has been described in a variety of non-human animal species including dogs, cats, pet reptiles, small ruminants, cattle, and poultry (Lepczyńska et al., 2017; Suwanti et al., 2020; Rauff-Adedotun et al., 2020; Rudzi´nska et al., 2020; Higuera et al., 2021; Shams et al., 2022) (**Supplementary Table S1**). The subtypes identified in the present study commonly circulate in other African countries with a few exceptions in which other countries have additional and different subtypes. For example, ST1, ST2, ST3, ST6 and ST7 have been identified in Nigeria (Poulsen et al., 2016) while ST1, ST2 and ST3 actively circulate in Sudan, Tanzania and Madagascar (Greigert et al., 2018; Cinek et al., 2021). Furthermore, ST1, ST2, ST3, ST5 and ST7 have been identified in Angola (Elena et al., 2018) while ST1, ST2, ST3, ST4 circulate in Mozambique (Muadica et al., 2021) and finally, evidence for the circulation of ST1, ST3, ST7, ST10 and ST14 in Senegal was also previously described (Khaled et al., 2020).

The four Blastocystis STs more frequently reported circulating in humans globally are ST1-ST4 (Alfellani et al., 2013). ST1, ST2, ST3 and ST6 which were also isolated in this study have also been isolated in humans (Graczyk et al., 2005; Yoshikawa et al., 2016) but are amongst the subtypes considered to have zoonotic potential (Cian et al., 2017; Mohammadpour et al., 2020) as they are commonly isolated in other mammals, primates, pigs and birds (Aynur et al., 2019). The finding of the zoonotic subtypes could be an indication of the spillover of infection from animals to humans (**Supplementary Table S1**). More specifically, the results of the present study revealed that some Zambian sequences clustered together with subtypes isolated elsewhere from nonhuman primates. Furthermore, whilst only a single sample of subtype ST6 was detected in our study, in Poland, ST6 has been shown to be zoonotic with domestic birds being reservoir hosts (Lewicki et al., 2016). In Brazil and Lebanon, ST6 also infects both humans and birds (Greige et al., 2018; Jiménez et al., 2019).

Therefore, considering that these animal species have overlapping interaction with human communities in Zambia, future studies should screen for the parasite in these animal hosts and in general livestock; more especially in areas with evidence of human colonization to further clarify the parasite epidemiology. Such information will help to establish whether the subtypes circulating in humans also circulate in animal hosts interacting with human and if indeed zoonotic transmission between these host species occurs.

ST1 was also reported at a high prevalence in this study despite it being commonly associated with other mammals. In a study in Libya, ST1 was reported to be the most predominant Blastocystis and this was ascribed to high animal ownership in that community, which allowed for transmission of the parasite from animals to humans (Abdulsalam et al., 2013). A high prevalence of ST1 has also been reported elsewhere (Deeb and Khodeer, 2013; Safadi et al., 2014), an indication of its common occurrence in humans. Blastocystis colonization

In conclusion, whilst Blastocystis does not appear to be a very significant health burden, the commensal does circulate in the country. There is suggestive evidence that both anthroponotic and zoonotic transmission could be playing an important role in the epidemiology of the country. There is thus need to collect and analyze environmental (e.g., water, fresh produce, soil) and animal (companion, livestock, and wildlife) samples to demonstrate the occurrence and genetic diversity of Blastocystis, so as to have a better understanding of the epidemiology. Further, more studies are needed to ascertain its pathogenic potential in humans. One limitation of this study was that most archival stool samples at the facility lacked corresponding definitive clinical diagnosis and epidemiological data. Therefore, future studies should take a longitudinal active surveillance approach, probably at country level, so that risk factors associated with the Blastocystis colonization and subsequent clinical manifestations in humans in Zambia are correctly determined.

## Data availability statement

The datasets presented in this study can be found in online repositories. The names of the repository/repositories and accession number(s) can be found in the article/**Supplementary Material**.

## Ethics statement

Ethical approval for the study was provided by the University of Zambia Biomedical Research Ethics Committee (UNZABREC) under reference number 1727-2021 while the authority to conduct research was given by the National Health Research Authority (NHRA) of Zambia, under reference number 00001/10/06/2021. Written informed consent from the patients/participants was not required to participate in this study in accordance with the national legislation and the institutional requirements.

## Author contributions

Conceived and designed the experiments (GM, KH, MMu); Data collection (GM, NM, RP, SS); Molecular laboratory investigation: (GM, NM, RP, SS, MMa); Supervised the research (KH, MMu); Data analysis (GM, KH, BM, ES, WM, SC); Drafting and proofreading of the manuscript (GM, KH, BM, ES, NM, RP, SS, MMa, WM, KC, SC, MMu). All authors contributed to the article and approved the submitted version.

## Funding

This study was supported by the Japan Agency for Medical Research and Development (grant no. JP21wm0125008). The funder had no role in the design of the study, data collection, analysis, decision to publish, or preparation of the manuscript.

## Acknowledgments

We would like to acknowledge the assistance of the laboratory technicians from the University Teaching Hospitals who were involved in the collection of samples used in this study.

## Conflict of interest

The authors declare that the research was conducted in the absence of any commercial or financial relationships that could be construed as a potential conflict of interest.

## Publisher’s note

All claims expressed in this article are solely those of the authors and do not necessarily represent those of their affiliated organizations, or those of the publisher, the editors and the reviewers. Any product that may be evaluated in this article, or claim that may be made by its manufacturer, is not guaranteed or endorsed by the publisher.
