# Supplementary material for: Molecular subtyping of Blastocystis sp. detected in patients at a large tertiary referral hospital in Lusaka, Zambia
Source: Front Parasitol. 2022 Oct 27;1:1033485. doi: 10.3389/fpara.2022.1033485 (PMC11731786; doi:10.3389/fpara.2022.1033485)
Supplement: Supplementary file 1 [file Table_1.docx]

**Table S1: Subtype and allele assignment for the *Blastocystis* sp. sequenced in the present study**

**GenBank number Sample ID ST      Allele**
LC639128                        ZAM01/2020 ST3      37
LC639129                        ZAM02/2020 ST1      4
LC639130                  ZAM05/2020 ST3      36
LC639131                        ZAM08/2020 ST3      38
LC639132                        ZAM09/2020 ST1      4
LC639133                        ZAM10/2020 ST3      37
LC639134                        ZAM11/2020 ST1      4
LC639135                        ZAM12/2020 ST2      12
LC639136                        ZAM14/2020 ST3      36
LC639137                        ZAM15/2020 ST3      39
LC639138                        ZAM16/2020 ST3      34
LC639139                        ZAM17/2020 ST1      4
LC639140                        ZAM19/2020 ST3      34
LC639141                        ZAM20/2020 ST3      36
LC639142                        ZAM26/2020 ST2      12
LC639143                        ZAM28/2020 ST3      36
LC639144                        ZAM29/2020 ST3      38
LC639145                        ZAM30/2020 ST1      4
LC639146                        ZAM31/2020 ST1      4
LC639147                        ZAM32/2020 ST2      12
LC639148                        ZAM33/2020 ST3      36
LC639149                        ZAM35/2020 ST2      12
LC639150                        ZAM36/2020 ST3      38
LC639151                        ZAM37/2020 ST2      12
LC639152                        ZAM39/2020 ST3      34
LC639153                        ZAM40/2020 ST3      37
LC639154                        ZAM41/2020 ST3      34
LC639155                        ZAM42/2020 ST2      9
LC639156                        ZAM44/2020 ST3      36
LC639157                        ZAM46/2020 ST3      34
LC639158                        ZAM52/2020 ST3      36
LC639159                        ZAM54/2020 ST1      4
LC639160                        ZAM58/2020 ST3      39
LC639161                        ZAM61/2020 ST1      4
LC639162                        ZAM62/2020 ST3      34
LC639163                        ZAM64/2020 ST3      38
LC639164                        ZAM66/2020 ST1      4
LC639165                        ZAM67/2020 ST1      4
LC639166                        ZAM73/2020 ST1      4
LC639167                        ZAM74/2020 ST3      38
LC639168                        ZAM80/2020 ST1      4
LC639169                        ZAM82/2020 ST2      12
LC639170                        ZAM83/2020 ST6      122
LC639171                        ZAM84/2020 ST3      38
LC639172                        ZAM85/2020 ST1      4
