# Supplementary material for: Molecular subtyping of Blastocystis sp. detected in patients at a large tertiary referral hospital in Lusaka, Zambia
Source: Front Parasitol. 2022 Oct 27;1:1033485. doi: 10.3389/fpara.2022.1033485 (PMC11731786; doi:10.3389/fpara.2022.1033485)
Supplement: Supplementary file 2 [file Table_2.docx]

**Table S2. *Blastocystis* subtypes identity based on the partial fragment of the 18S SSU-rDNA**

| **DNA ID** | **Reference Strains of *Blastocystis* species** | **GenBank Accession Number** | **Nucleotide percent identity (%)** |
| --- | --- | --- | --- |
| 1 | *Blastocystis* sp. Isolate 33F small subunit ribosomal RNA gene, partial sequence. | [MN686282.1](https://www.ncbi.nlm.nih.gov/nucleotide/MN686282.1?report=genbank&log$=nucltop&blast_rank=1&RID=W1ENM5EG013) | 100 |
| 9 | *Blastocystis* sp. Isolate 587 small subunit ribosomal RNA gene, partial sequence. | [MN585822.1](https://www.ncbi.nlm.nih.gov/nucleotide/MN585822.1?report=genbank&log$=nucltop&blast_rank=1&RID=W1ES4FH0013) | 99.83 |
| 5 | *Blastocystis* sp. Strain ST3 voucher S245-DYS small subunit ribosomal RNA gene, partial sequence. | [MK719673.1](https://www.ncbi.nlm.nih.gov/nucleotide/MK719673.1?report=genbank&log$=nucltop&blast_rank=1&RID=W1EY99H701N) | 100 |
| 2 | *Blastocystis* sp. Isolate C16_MELOGB small subunit ribosomal RNA gene, partial sequence. | [MK511793.1](https://www.ncbi.nlm.nih.gov/nucleotide/MK511793.1?report=genbank&log$=nucltop&blast_rank=1&RID=W1F157RK013) | 99.66 |
| 10 | *Blastocystis* *hominis* Isolate B195 small subunit ribosomal RNA gene, partial sequence. | M[T042791.1](https://www.ncbi.nlm.nih.gov/nucleotide/MT042791.1?report=genbank&log$=nucltop&blast_rank=1&RID=W1F614JK013) | 99.83 |
| 14 | *Blastocystis* sp. Strain ST3 voucher S245-DYS small subunit ribosomal RNA gene, partial sequence. | [MK719673.1](https://www.ncbi.nlm.nih.gov/nucleotide/MK719673.1?report=genbank&log$=nucltop&blast_rank=1&RID=W1FP5TEE01N) | 100 |
| 11 | *Blastocystis* sp. Isolate 36M small subunit ribosomal RNA gene, partial sequence. | [MN658568.1](https://www.ncbi.nlm.nih.gov/nucleotide/MN658568.1?report=genbank&log$=nucltop&blast_rank=1&RID=W2162FYR016) | 99.49 |
| 15 | *Blastocystis* sp. Clone 34 small subunit ribosomal RNA gene, partial sequence. | [MN526781.1](https://www.ncbi.nlm.nih.gov/nucleotide/MN526781.1?report=genbank&log$=nucltop&blast_rank=1&RID=W21HTA1F016) | 99.83 |
| 8 | *Blastocystis* *hominis* Isolate B99 small subunit ribosomal RNA gene, partial sequence. | [MT042787.1](https://www.ncbi.nlm.nih.gov/nucleotide/MT042787.1?report=genbank&log$=nucltop&blast_rank=1&RID=W21MWZ9H016) | 100 |
| 12 | *Blastocystis* *homonis* Isolate B113 small subunit ribosomal RNA gene, partial sequence. | [MT042820.1](https://www.ncbi.nlm.nih.gov/nucleotide/MT042820.1?report=genbank&log$=nucltop&blast_rank=1&RID=W21TMMDX013) | 99.83 |
| 26 | *Blastocystis* sp. Clone 5 small subunit ribosomal RNA gene, partial sequence. | M[N526752.1](https://www.ncbi.nlm.nih.gov/nucleotide/MN526752.1?report=genbank&log$=nucltop&blast_rank=1&RID=W21YNA66013) | 99.83 |
| 28 | [*Blastocystis* sp. strain ST 3 voucher S245-DYS small subunit ribosomal RNA gene, partial sequence](https://blast.ncbi.nlm.nih.gov/Blast.cgi#alnHdr_1604405446) | [MK719673.1](https://www.ncbi.nlm.nih.gov/nucleotide/MK719673.1?report=genbank&log$=nucltop&blast_rank=1&RID=W22226JE01N) | 100 |
| 37 | *Blastocystis* *hominis* Isolate B113 small subunit ribosomal RNA gene, partial sequence. | [MT042820.1](https://www.ncbi.nlm.nih.gov/nucleotide/MT042820.1?report=genbank&log$=nucltop&blast_rank=1&RID=W22A6RZ7013) | 99.82 |
| 40 | *Blastocystis* sp. Strain ST3 voucher ASYM-310 small subunit ribosomal RNA gene, partial sequence. | [MK719661.1](https://www.ncbi.nlm.nih.gov/nucleotide/MK719661.1?report=genbank&log$=nucltop&blast_rank=1&RID=W1A0PGAN016) | 99.83 |
| 41 | *Blastocystis* sp. Subtype 3 isolate B66BAS small subunit ribosomal RNA gene, partial sequence. | [KY610149.1](https://www.ncbi.nlm.nih.gov/nucleotide/KY610149.1?report=genbank&log$=nucltop&blast_rank=1&RID=W1A6M31E013) | 100 |
| 42 | *Blastocystis* sp. Isolate Br68 small subunit ribosomal RNA gene, partial sequence. | [MG807911.1](https://www.ncbi.nlm.nih.gov/nucleotide/MG807911.1?report=genbank&log$=nucltop&blast_rank=1&RID=W1CX1TAW01N) | 100 |
| 44 | *Blastocystis* sp. Strain ST3a | MT645665 | 100 |
| 46 | [*Blastocystis hominis* isolate B195 small subunit ribosomal RNA gene, partial sequence](https://blast.ncbi.nlm.nih.gov/Blast.cgi#alnHdr_1808209492) | [MT042791.1](https://www.ncbi.nlm.nih.gov/nucleotide/MT042791.1?report=genbank&log$=nucltop&blast_rank=1&RID=W1DXG0A201N) | 100 |
| 48 | *Blastocystis*sp. Clone 5 | MN 526752 | 99.83 |
| 49 | *Blastocystis*sp. Strain ST3 | MK719673 | 100 |
| 50 | *Blastocystis*sp. Isolate 33F | MN686282 | 100 |
| 52 | *Blastocystis hominis*isolate 356_3 | MT042797 | 100 |
| 53 | *Blastocystis sp.*Isolate C29 | MN585864 | 99.83 |
| 54 | *Blastocystis sp.*Isolate C29 | MN585864 | 100 |
| 55 | *Blastocystis sp.*Isolate C29 | MN585864 | 99.83 |
| 57 | *Blastocystis sp.*Subtype 1 strain PAN12 | JQ974923 | 98.45 |
| 58 | *Blastocystis hominis*isolate B99 | MT042787 | 99.74 |
| 60 | *Blastocystis*sp. Clone 34 | MN526781 | 100 |
| 61 | *Blastocystis*sp. Isolate C29 | MN585864 | 100 |
| 62 | *Blastocystis*sp. Strain ST3 | MK719673 | 100 |
| 63 | *Blastocystis*sp. Strain ST3 | MT645669 | 99.64 |
| 64 | *Blastocystis*sp. Clone 34 | MN526781 | 100 |
| 65 | *Blastocystis*sp. Strain ST3 | MT645669 | 97.48 |
| 66 | *Blastocystis hominis*isolate B9 | MT042818 | 100 |
| 67 | *Blastocystis* sp. Isolate C14 small subunit ribosomal RNA gene, partial sequence. | [MN585857.1](https://www.ncbi.nlm.nih.gov/nucleotide/MN585857.1?report=genbank&log$=nucltop&blast_rank=1&RID=W1EEN42R013) | 99.66 |
| 69 | *Blastocystis*sp. Subtype 3 | KY610153 | 100 |
| 70 | *Blastocystis*sp. Clone 65 | MN526812 | 100 |
| 73 | *Blastocystis*sp. Subtype 1 | KY190413 | 100 |
| 74 | *Blastocystis*sp. Clone 34 | MN526781 | 100 |
| 77 | *Blastocystis*sp. Subtype 1 | MG011609 | 100 |
| 78 | *Blastocystis hominis*isolate B195 | MT042791 | 99.83 |
| 79 | *Blastocystis*sp. Strain ST3 | MK719673 | 99.47 |
| 80 | *Blastocystis*sp. Isolate C29 | MN585864 | 99.66 |
| 81 | *Blastocystis*sp. Isolate 13UC_2ST3 | MK782504 | 100 |
| 82 | *Blastocystis*sp. Isolate 22F | MN686280 | 100 |
| 83 | *Blastocystis*sp. Isolate 1104 | MN585849 | 100 |
| 84 | *Blastocystis*sp. Clone 34 | MN526781 | 100 |
| 85 | *Blastocystis* sp. Isolate MC17 small subunit ribosomal RNA gene, partial sequence. | [MH197669.1](https://www.ncbi.nlm.nih.gov/nucleotide/MH197669.1?report=genbank&log$=nucltop&blast_rank=1&RID=W1E4DD6M013) | 100 |
